# Diverse mechanisms for spliceosome-mediated 3′ end processing of telomerase RNA

**DOI:** 10.1038/ncomms7104

**Published:** 2015-01-19

**Authors:** Ram Kannan, Rachel M. Helston, Richard O. Dannebaum, Peter Baumann

**Affiliations:** 1 Stowers Institute for Medical Research, Kansas City, Missouri 64110, USA; 2 Department of Molecular and Integrative Physiology, University of Kansas Medical Center, Kansas City, Kansas 66160, USA; 3 Howard Hughes Medical Institute, Kansas City, Missouri 64110, USA

## Abstract

The 3′ end of *Schizosaccharomyces pombe* telomerase RNA (*Sp*TER1) is generated by spliceosomal cleavage, a reaction that corresponds to the first step of splicing. The observation that the spliceosome functions in 3′ end processing raised questions about the evolutionary origin and conservation of this mechanism. We now present data in support of spliceosomes generating 3′ ends of telomerase RNAs in other fungi. Strikingly, the mechanistic basis for restricting spliceosomal splicing to the first transesterification reaction differs substantially among species. Unlike *S. pombe*, two other fission yeasts rely on hyperstabilization of the U6 snRNA—5′ splice site interaction to impede the 2nd step of splicing. In contrast, a non-canonical 5′ splice site blocks the second transesterification reaction in *Aspergillus* species. These results demonstrate a conserved role for spliceosomes functioning in 3′ end processing. Divergent mechanisms of uncoupling the two steps of splicing argue for multiple origins of this pathway.

## Abstract (web summary)

In fission yeast, the telomerase RNA (TER) is produced through spliceosomal cleavage. Here, Kannan *et al*. find that spliceosome-generated 3′ ends also occurs in other fungal TERs using distinct molecular mechanisms, suggesting multiple origins for this type of TER maturation pathway.

## Graphical abstract

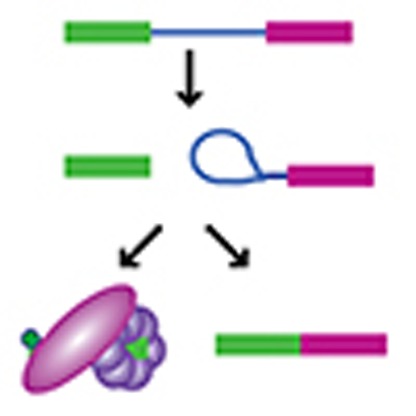

Telomerase is the ribonucleoprotein (RNP) complex that synthesizes telomeric repeats and maintains telomere length homeostasis. The catalytic core of telomerase is composed of a non-coding RNA (TER) that contains a template region and a protein subunit (TERT) that reiteratively reverse transcribes the template to add telomeric repeats onto chromosome ends^1^. Insufficient telomerase activity is causative in a number of degenerative syndromes where premature depletion of stem cell pools results in pleiotropic phenotypes, including nail dystrophy, aplastic anaemia and pulmonary fibrosis^2^. Conversely, telomerase is active in the majority of cancer cells and is required for their continued proliferation^3^. Telomerase inhibition may thus constitute a promising avenue in cancer treatment. While much has been learned over the past decade about the regulation of telomerase expression and activity, we know much less about the biogenesis of the RNP^4^.

The fission yeast *Schizosaccharomyces pombe* has served as a valuable model system for studying telomerase. The identification of human TERT and Pot1 proteins was aided by the prior characterization of the fission yeast homologues^5^,^6^, and analysis of telomerase biogenesis in *S. pombe* has elucidated an ordered sequence of events that culminate in the assembly of the functional RNP^7^,^8^. The extent to which these events are conserved in other species remains to be determined, but key aspects including cap hypermethylation and association with Sm proteins have been reported for human telomerase as well^9^,^10^. The *S. pombe* telomerase RNA subunit TER1 (refs 11, 12) is transcribed as a precursor harbouring an intron immediately downstream of its mature 3′ end^7^. Instead of the intron being removed by two consecutive transesterification reactions, the spliceosome only carries out the first cleavage reaction and releases the 5′ exon to become the functional form of telomerase RNA.

In the first step of splicing, the 2′ hydroxyl group of the branch point (BP) adenosine, located within the branch site (BS) sequence, attacks the sugar–phosphate bond at the 5′ splice site (5′SS), forming a 2′–5′ linkage and producing a free 5′ exon and a branched species, the lariat intermediate. During intron excision, this first reaction is followed by a conformational rearrangement that brings the now exposed 3′ hydroxyl of the upstream exon into close proximity with the 3′ splice site (3′SS). A second transesterification reaction yields ligated messenger RNA (mRNA) and the lariat form of the intron. The release of the splicing intermediate as a functional product in the case of *Sp*TER1 is triggered by an unusual combination of two intronic features—a long distance between the BP and 3′SS and a BS sequence that is fully complementary to the binding site in U2 small nuclear RNA (snRNA)^13^. These features attenuate the transition from first- to second-step conformation and result in the release of the splicing intermediates. Mechanistically, this process is akin to ‘discard’, a pathway that has been characterized through a series of elegant experiments in budding yeast where the DExD/H-box helicase Prp22 functions in the recovery of spliceosomes that have selected suboptimal 3′ splice sites^14^,^15^. Up to this point, *Sp*TER1 has been the only known example of the discard pathway generating a functional product.

The recent discovery of the Nrd1/Nab3 pathway mediating 3′ end formation for telomerase RNA in budding yeast^16^,^17^ has raised the question whether spliceosomal cleavage as a mechanism of 3′ end processing is restricted to *S. pombe*. Here we show that telomerase RNAs from two other fission yeasts and from filamentous fungi contain introns that undergo spliceosomal cleavage. Interestingly, while the use of the first step of splicing to generate telomerase RNA 3′ ends is conserved, the RNA elements that uncouple the two steps of splicing are entirely different.

## Results

To examine whether the mechanism of telomerase RNA 3′ end processing is conserved across fission yeast species, we identified the telomerase RNA subunits from *S. cryophilus* and *S. octosporus* based on synteny in the corresponding regions of the genomes followed by alignment with *Sp*TER1. Pairwise alignments revealed substantial sequence divergence among the RNA subunits, consistent with previous reports that telomerase RNAs evolve rapidly^18^,^19^,^20^. It is important to note that the genus *Schizosaccharomyces* encompasses species that are as divergent as humans and amphioxus^21^. Even the two most closely related species *S. cryophilus* and *S. octosporus* are as distantly related as humans and dogs. The template region, which is generally more highly conserved among telomerase RNAs, differs between *S. pombe* and the other fission yeast species, with *S. cryophilus* and *S. octosporus* sequences containing a three-nucleotide insertion relative to *S. pombe* (Supplementary Fig. 1). When this sequence was inserted into *S. pombe* TER1 and telomeres were cloned and sequenced, near-perfect GGGTTACTT repeats were added to chromosome ends rather than the heterogeneous G_2–6_TTACA_0–1_C_0–1_ repeat generated by the endogenous template (Supplementary Fig. 2). Examination of whole-genome sequencing data for *S. cryophilus* and *S. octosporus* by the tandem repeat finder identified the same perfect repeat as a probable candidate telomere sequence for both species^21^.

**Figure 1:**
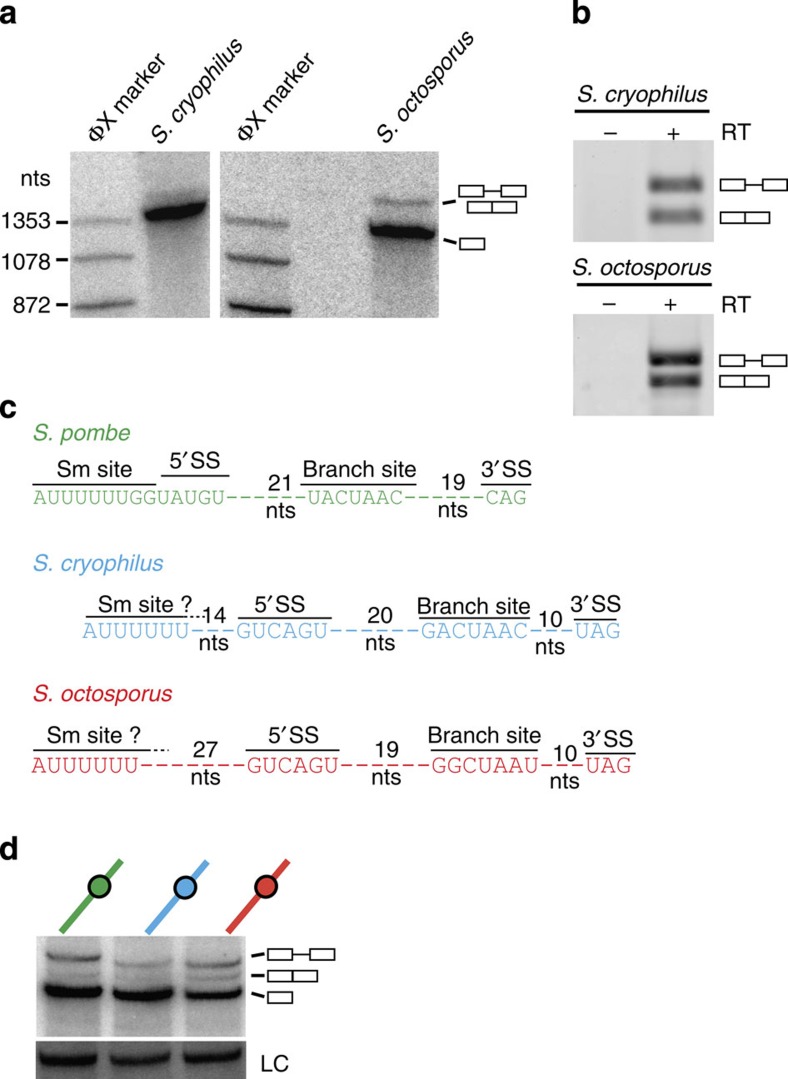
Identification and characterization of telomerase RNA from *Schizosaccharomyces cryophilus* and *S. octosporus*. (**a**) Northern blot for telomerase RNA from *S. cryophilus* (left) and *S. octosporus* (right). Total RNA of the respective species was separated on a 4% polyacrylamide gel and probed with ^32^P-labelled fragments corresponding to the first exon of the respective TER1 gene. (**b**) RT–PCR using primer pairs spanning the putative introns in *Sc*TER1 and *So*TER1. The identities of the upper band as unspliced and lower band as spliced products were verified by sequencing. (**c**) Schematic of the introns located downstream of the 3′ ends of the mature forms in each of the three species. Distances between the Sm-binding site, 5′ splice site, branch site and 3′ splice site are given in nucleotides (nts). Designation of the Sm-/LSm-binding sites in *S. cryophilus* and *S. octosporus* is based on sequence similarity, not on experimental verification of Sm/LSm binding. To indicate this fact, the sites are labelled with a question mark. (**d**) RNaseH cleavage followed by northern blot visualizes the relative abundance of precursor, spliced and cleaved forms of *Sp*TER1 containing the respective introns (colours as in **c**). An oligonucleotide probe against the snoRNA sn101 was used as a loading control.

Probes for the presumed TER1 transcripts identified a prominent band migrating at ~1,350 nucleotides in total RNA samples from *S. cryophilus* (Fig. 1a). Due to the size heterogeneity resulting from multiple polyadenylation sites and variable poly(A) tail length, it is not unusual for the telomerase RNA precursor to run as a smear on polyacrylamide gels. Two bands were observed for *S. octosporus*, reminiscent of the precursor and mature form described for *S. pombe*^12^. Mapping the 5′ and 3′ ends of these RNAs by circular reverse-transcription (RT)–PCR revealed further similarities as well as differences from *S. pombe*. The major forms of *S. cryophilus* and *S. octosporus* TER1 are 1,291 and 1,270 nucleotides in length (Supplementary Fig. 1) compared with 1,213 nucleotides for *S. pombe*^22^. Longer polyadenylated forms were also detected, as well as putative introns located downstream of the 3′ end of the major forms. RT–PCR from RNA samples of the respective species confirmed the presence of unspliced and spliced forms, indicative of a conserved role for the spliceosome in telomerase RNA maturation (Fig. 1b).

Unlike *S. pombe* where the Sm-protein-binding site and a 5′ splice site overlap by one nucleotide, 5′ splice sites were found 14 (*S. cryophilus*) and 27 (*S. octosporus*) nucleotides downstream of the 3′ ends of the mature forms, which fall within putative Sm-/LSm-binding sites (Fig. 1c). Detection of several clones that terminate at sites between the 5′SS and the Sm site suggest that spliceosomal cleavage is followed by exonucleolytic degradation until binding by the LSm2–8 complex stabilizes the 3′ end, as shown for *S. pombe*^8^.

In light of the critical importance of a strong BS and long BP—3′SS distance for spliceosomal cleavage in *S. pombe*, we were surprised to find that the BS sequences did not correspond to the perfect complement of the binding sequence in U2 snRNA nor was the distance between BP and 3′SS unusually long (Fig. 1c). We therefore wondered whether 3′ end formation in *S. cryophilus* and *S. octosporus* even involved the release of the 5′ exon after the first cleavage reaction, or whether complete splicing is followed by exonucleolytic degradation.

### Spliceosomal cleavage is conserved among fission yeasts

**Figure 2:**
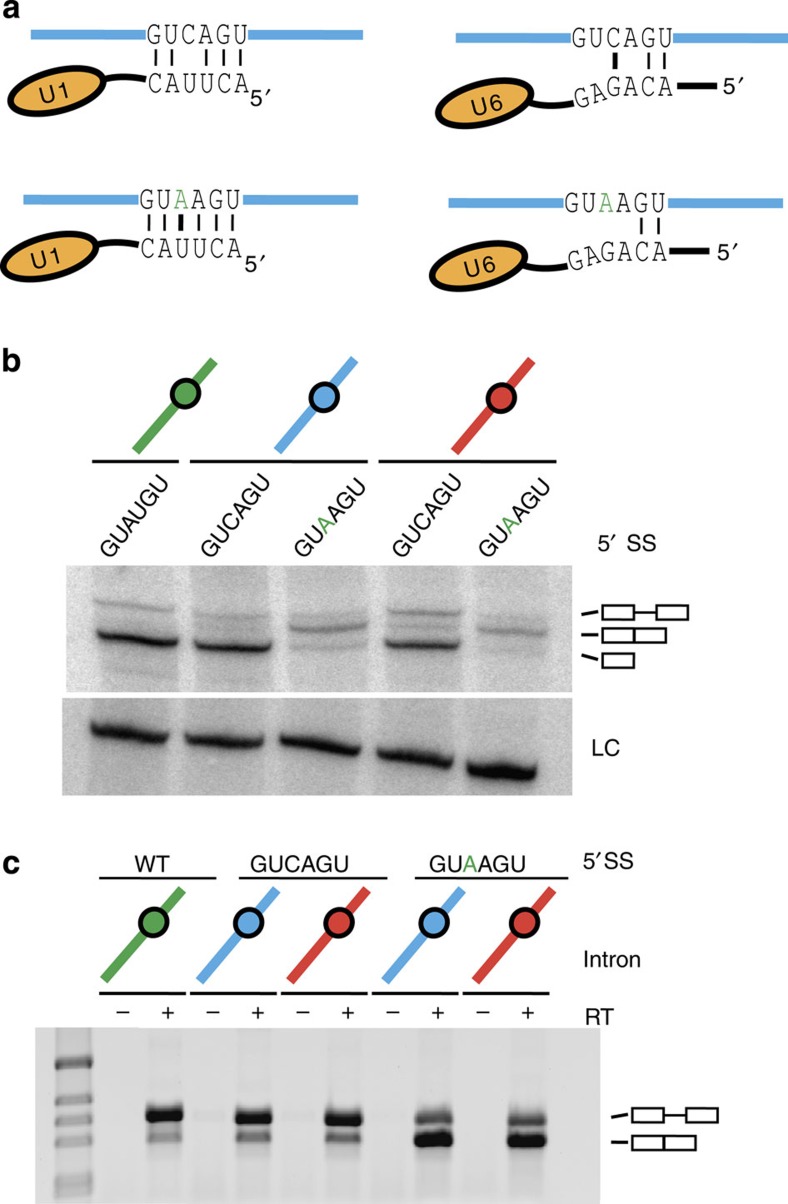
Hyperstabilization of the 5′SS:U6 snRNA interaction impedes completion of splicing for the introns from *S. cryophilus* and *S. octosporus*. (**a**) Schematic of interactions between the 5′SS found in *S. octosporus* and *S. cryophilus* TER1 and U1 and U6 snRNA, respectively. The single-nucleotide change C3 to A (indicated in green) adds an A:U interaction for U1, but destabilizes the interaction with U6 snRNA. (**b**) RNaseH cleavage followed by northern blot to visualize precursor, cleaved and spliced forms of TER1 containing the intron from *S. pombe* (green), *S. cryophilus* (blue) and *S. octosporus* (red) or mutant versions thereof. (**c**) RT–PCR visualizing relative abundance of precursor and spliced forms for the different constructs. LC, loading control.

Replacement of the *S. pombe* TER1 intron with introns from protein-encoding genes, which lack the combination of features that promote release after the first step, results in complete splicing of TER1 (ref. 7). In contrast, when the introns of *S. cryophilus* and *S. octosporus* TER1 were introduced into *Sp*TER1, the mature form of TER1 was readily produced (Fig. 1d). Similar to wild-type *Sp*TER1, only a small amount of the spliced form was observed by RT–PCR (Fig. 2c). In summary, despite the absence of all features previously defined as critical for spliceosomal 3′ end processing, the introns of *S. cryophilus* and *S. octosporus* TER1 predominantly undergo the first step of splicing followed by release of the 5′ exon.

To understand why splicing fails to go to completion for these introns, we searched for features that distinguish the *Sc* and *So*TER1 sequences from introns in protein-encoding genes. Intron length, BP to 3′SS distance, BS and 3′SS sequence were all similar to the consensus derived from annotated introns in the respective species (data not shown). However, the 5′SS sequence in *Sc* and *So*TER1 was found to be highly unusual (Fig. 1c). Only 3 of 5,702 introns in *S. cryophilus* and 1 out of 5,350 annotated introns in *S. octosporus* contain the 5′SS sequence GUCAGU indicative of negative selection (Supplementary Fig. 3). This is likely significant in the context of TER1 introns undergoing the first, but not the second step of splicing. In budding yeast, studies of the dynamic interactions that occur between snRNAs and intronic sequences during splicing have shown that splicing efficiency is the outcome of kinetic competition between different conformations^23^,^24^. Among the mutations found to inhibit the second step of splicing were those that hyperstabilize the 5′SS:U6 snRNA interaction^25^.

To test whether the C at the +3 position was responsible for uncoupling the two steps of splicing in the context of TER1 processing, we introduced a C3A mutation, thereby destabilizing the interaction with U6 snRNA (Fig. 2a). Following this single-nucleotide change, the 5′SS now matches the most common 5′ splice site sequence in *S. cryophilus* and *S. octosporus* (Supplementary Fig. 3). This single-nucleotide change had a profound effect on TER1 processing, with the spliced form now accumulating at the expense of the cleaved form (Fig. 2b,c). These observations indicate that the spliceosome has a conserved function in generating the 3′ ends of telomerase RNA in diverse fission yeasts. Perhaps more surprisingly, the element that uncouples the two steps of splicing is entirely different from those in *S. pombe* TER1.

### 3′ end processing of telomerase RNA in filamentous fungi

**Figure 3:**
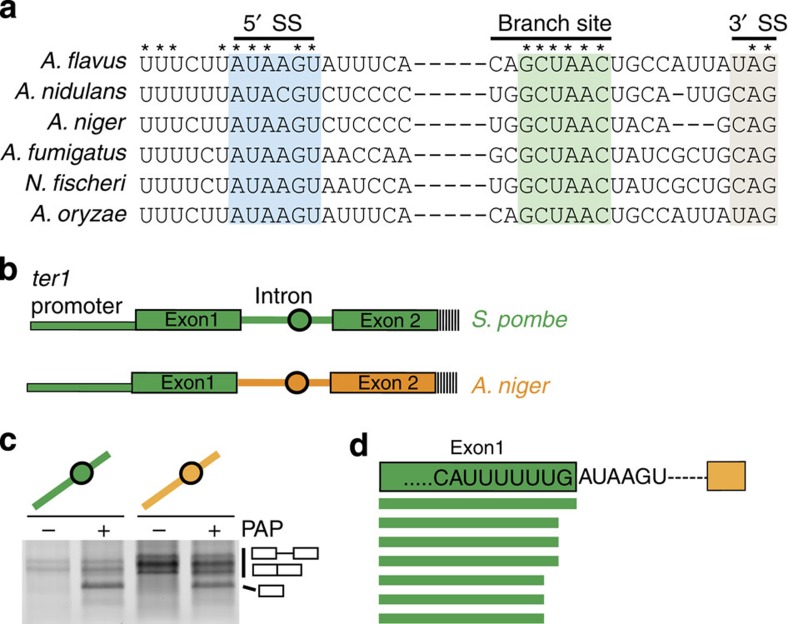
A non-canonical 5′ splice site permits spliceosomal cleavage during telomerase RNA biogenesis in filamentous fungi. (**a**) Partial alignment of telomerase RNAs from five *Aspergillus* species and *Neosartorya fischeri* generated in ClustalW2. Asterisks mark positions conserved across all six species. The conserved 5′SS, branch site and 3′SS are highlighted in blue, green and grey, respectively. (**b**) Schematic of constructs used to examine processing of *A. niger* sequences (orange) in the context of SpTER1 (green). (**c**) Untreated and poly(A) polymerase (PAP)-treated total RNA was subjected to reverse transcription in the presence of oligo dT followed by PCR to clone the 3′ ends of naturally polyadenylated (minus PAP lanes) and non-polyadenylated forms (product only observed in +PAP lanes). (**d**) Position of 3′ ends of the non-polyadenylated (cleaved) form of TER1 containing the intron and downstream sequence from *A. niger*.

To determine whether telomerase 3′ end processing by the spliceosome is more widely conserved, we turned our attention to the recently identified TER1 sequences from *Aspergilli* where the presence of a non-canonical, putative 5′SS had been noted^26^. The sequence AUA(A/C)GU, as well as putative branch and 3′ splice sites are found downstream of a pyrimidine-rich motif that resembles an Sm-binding site (Fig. 3a). Mutation of the highly conserved first nucleotide in an intron from G to A blocks the second step of splicing for introns from budding yeast and humans^27^,^28^,^29^. However, no example of an AU intron functioning in 3′ end processing had been reported thus far.

**Figure 4:**
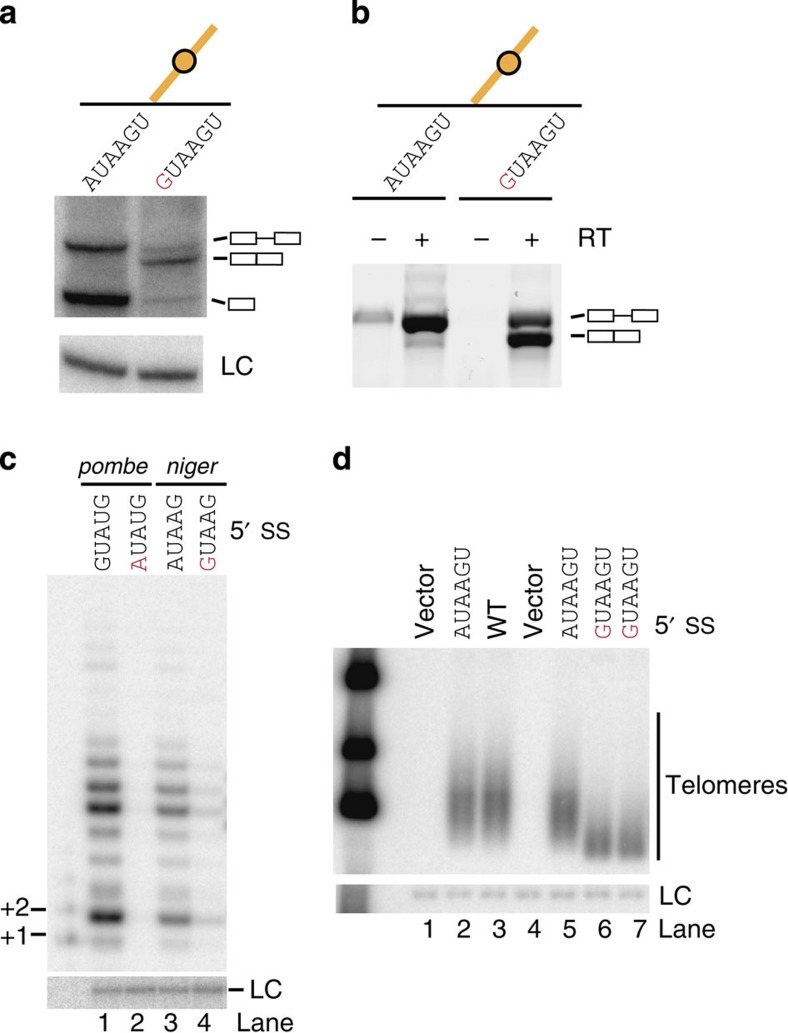
The non-canonical 5′ splice site blocks completion of splicing and promotes spliceosomal cleavage of the *A. niger* TER1 intron. (**a**) Northern blot on total RNA from cells expressing the wild-type *A. niger* intron or the A1 to G mutation shown in red. (**b**) RT–PCR visualizing precursor and spliced forms. (**c**) Telomerase activity assay using extracts from cells expressing telomerase RNA containing wild-type or mutant versions of the introns from *S. pombe* or *A. niger*, respectively. Wild-type sequence of 5′SS shown in black, mutated nucleotides in red. (**d**) Telomere length determined by Southern blotting of EcoRI-digested genomic DNA. Vector denotes the absence of telomerase RNA (biological replicates in lanes 1 and 4, WT denotes the wild-type version of *S. pombe* TER1 (lane 3), AUAAGU denotes the wild-type version of the *A. niger* intron (biological replicates in lanes 2 and 5) and GUAAGU denotes a mutated 5′SS in the *A. niger* intron (biological replicates in lanes 6 and 7). A 300-bp telomeric DNA fragment was used as a template for nick-translation to generate a ^32^P-labelled probe; a second probe for the *rad16* gene was used as a loading control (LC).

To assess whether the *Aspergillus* sequences can function in telomerase 3′ end processing, we replaced the TER1 intron and second exon with the corresponding sequence from *A. niger* (Fig. 3b). The mature, non-polyadenylated form of TER1 was readily detected by 3′ end cloning from cells harbouring the *A. niger* sequence (Fig. 3c). Sequencing revealed that these RNAs terminate within the Sm-binding site (Fig. 3d). Reminiscent of wild-type *S. pombe* TER1, the product of the first step of splicing is by far the most abundant form detected by northern analysis (Fig. 4a). Consistent with the adenine at the first position of the intron blocking the second step, the spliced form is not observed by northern blot and is only barely detected by RT–PCR across the intron (Fig. 4b).

We next mutated the A at the first position of the intron to G to ascertain whether this non-canonical 5′SS was indeed responsible for blocking the second step of splicing. This single-nucleotide change resulted in a >10-fold reduction in the cleaved form (Fig. 4a). Spliced product was now the dominant form of the RNA detected by RT–PCR (Fig. 4b). When telomerase activity was examined in extracts prepared from cells harbouring TER1 with the *A. niger* intron or an A1G mutant version, the former showed near-wild-type levels of activity (Fig. 4c, compares lanes 1 and 3). In contrast, telomerase activity was barely detectable for the A1G mutant (lane 4). Similarly, a 5′SS mutant in the *S. pombe* TER1 intron, which blocks the first step, reduced telomerase activity to below the threshold of detection (lane 2).

Consistent with the results from *in vitro* telomerase assays, the wild-type *A. niger* sequence fully rescued the maintenance of wild-type telomere length, whereas cells harbouring the A-to-G point mutant had shorter telomeres (Fig. 4d). These results demonstrate that the non-canonical 5′SS found near the 3′ end of telomerase RNA from *Aspergillus* species is necessary to trigger spliceosomal cleavage and can thus function in 3′ end processing of telomerase RNA. The result further demonstrates that 3′ end cleavage, but not the underlying mechanism, is critical for generating functional telomerase.

### Context-dependent effects of variant 5′ splice sites

**Figure 5:**
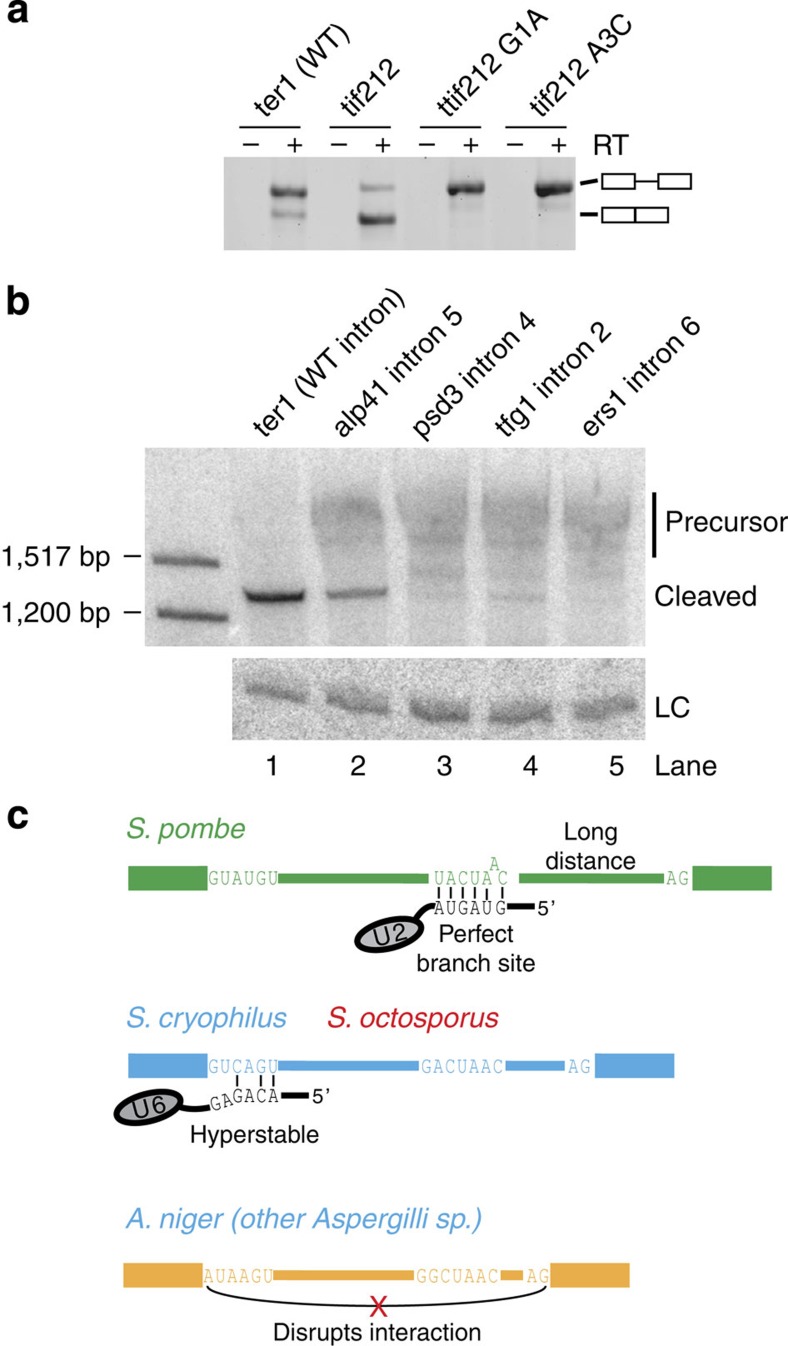
The effects of non-canonical 5′ splice sites are context dependent. (**a**) RT–PCR for the tif212 intron and 5′SS mutations in the context of TER1. (**b**) Northern blot for TER1 containing introns from protein-encoding genes with non-canonical 5′SS (GUCAGU, lanes 2–4; GUCUGU, lane 5). (**c**) Schematic of mechanisms that promote spliceosomal cleavage of telomerase RNA in different organisms. LC, loading control.

Considering that adenosine in intron position 1 or cytosine in position 3 are exceedingly rare among annotated introns, we asked whether these deviations from a consensus 5′SS are by themselves sufficient to trigger spliceosomal cleavage. We had previously introduced the G1A mutation into the *S. pombe* TER1 intron and found that it blocked the first step of splicing^7^. We now introduced this mutation in the context of an intron from the protein-encoding *tif212* gene, which also has the 5′SS sequence GUAUGU. As cleavage products of the mRNAs may be unstable, we performed these experiments in the context of TER1 exonic sequences, where we had previously demonstrated that LSm2–8 binding stabilizes the cleaved product^8^. The +1A mutation inhibited splicing (Fig. 5a) and polyadenylation was observed to occur at a variety of sites within the intron (Supplementary Fig. 4A), consistent with U1 binding inhibiting polyadenylation nearby^30^. Splicing was also inhibited by the +3C mutation, but most clones terminated downstream of the intron (Supplementary Fig. 4B). A few clones ending at the Sm-binding site were observed, but based on the scattered distribution of 3′ ends these are more likely to be the result of exonucleolytic degradation of the precursor than spliceosomal cleavage. We thus conclude that in the context of the tif212 intron, the A3C mutation inhibits spliceosome assembly, or the first step.

We next introduced three naturally occurring *S. pombe* introns with GUCAGU and one with a GUCUGU 5′SS sequence into TER1 to ascertain whether these introns are subject to spliceosomal cleavage. Similar to the endogenous TER1 intron, only small amounts of the spliced product were observed with each of these introns (Supplementary Fig. 5A). Although the polyadenylated precursor was the dominant form based on northern blots, cleaved forms were indeed observed and for intron 5 from the alp41 gene amounted to 40% of the cleaved RNA seen with the native TER1 intron (Fig. 5b). Cloning and sequencing of 3′ ends confirmed the presence of cleaved products terminating at the Sm-binding site for each intron (Supplementary Fig. 5B). In summary, both +3C and +1A promote spliceosomal cleavage in a context-dependent manner, but are by themselves insufficient to promote spliceosomal cleavage to the extent observed in telomerase RNA introns.

## Discussion

The observation that the spliceosome has a role in 3′ end processing of telomerase RNA in *S. pombe* came as a surprise at first. Subsequent mechanistic studies revealed that release of cleaved RNA after the first transesterification reaction requires a strong BS and a long distance between the BP and 3′SS^13^. Here we demonstrate that despite these features being absent from telomerase RNAs in other fission yeasts and filamentous fungi, the role of the spliceosome in telomerase RNA 3′ end formation is nevertheless conserved. The evolutionary implication of these findings is that the ancestral telomerase RNA subunit must have contained an intron that was spliced during biogenesis. At least three independent mechanisms have emerged to convert splicing into 3′ end processing by aborting the process after the first cleavage reaction.

It has long been appreciated that the spliceosome is a highly dynamic machine, which undergoes several compositional and conformational changes during the course of a splicing reaction. Comprehensive genetic and biochemical analyses of mutations in intron sequences and splicing factors, and in particular examination of compensatory effects, have resulted in the ‘two-state model’, which postulates that conformations supporting the two catalytic reactions are in kinetic competition with each other^23^,^31^,^32^. Numerous mutations in the RNA substrate, as well as in the protein and RNA components of the spliceosome, have been found to stabilize one conformation over the other thereby affecting the outcome of the reaction. These studies, combined with examination of telomerase RNA processing in different species, now suggest a common basis for how the spliceosome functions in 3′ end processing:

In *S. pombe*, the transition from the first to the pre-second-step conformation is slow due to the long distance between BP and 3′SS, and the perfect sequence complementarity between the BS and the BS-binding sequence in U2 snRNA (Fig. 5c). Consequently, the splicing intermediates are discarded via a kinetic proofreading mechanism involving the DExD/H-box helicases Prp22 and Prp43 (refs 13, 15). Several other mutations have been described in the splicing literature that trigger discard by attenuating the transition to the pre-second-step conformation or by blocking second-step catalysis, including changes of conserved nucleotides at the 5′SS, BS and 3′SS. We now show that at least two of these sequence variants occur naturally in RNA substrates that undergo spliceosomal cleavage as part of telomerase RNA biogenesis.

In the fission yeasts *S. cryophilus* and *S. octosporus*, the cytosine at the third position of the highly unusual 5′SS sequence GUCAGU provides the opportunity for an additional G:C base pair with U6 snRNA, thereby hyperstabilizing the 5′SS:U6 interaction (Fig. 5c). Extensive mutational analysis in the context of the budding yeast actin intron demonstrated that a hyperstabilized 5′SS:U6 duplex becomes rate limiting for the transition between the first and second step^25^. In this case, GUCUGU inhibited the second step, whereas GUCAGU, which lacks the ability to base pair at position 4, did not. It appears that the fission yeast spliceosome is more sensitive with regards to 5′SS:U6 stability at least in the context of TER1 introns, since the GUCAGU sequence inhibits the second step for several introns examined here. We have previously shown that GUCUGU and GUCAGU both inhibit the first step when introduced into the *S. pombe* TER1 intron^13^, further illustrating that the effects of splice site sequence variations are highly context dependent.

In the case of *Aspergillus sp.*, a non-canonical 5′ splice site AU is critical for limiting the spliceosome to carrying out the first reaction followed by the release of the cleaved products. On the basis of covariation of the first and last nucleotide of introns and the reciprocal suppression of mutations at these positions, it has been proposed that a non-Watson–Crick interaction between the first and last guanosine is pivotal to position the 3′ splice site for the second cleavage reaction^33^. The adenine at position 1 of the TER1 intron in *Aspergillus* blocks this interaction and promotes release of the products after the first cleavage reaction (Fig. 5c).

The realization that several different sequence variations can attenuate or block the transition to the second-step conformation has hampered computational attempts to find other RNAs that undergo spliceosomal cleavage as a mechanism of 3′ end maturation. It appears that processing of telomerase RNA by spliceosomal cleavage is under positive selection in diverse fungi, but the underlying mechanism is not. With evidence for three distinct means of attenuating the transition to the second step being critical for telomerase RNA 3′ end processing in different species, it is tempting to speculate that additional mechanisms underlying 3′ end processing by the spliceosome will be identified in due course. For example, a guanosine at the BP or an AG to AC mutation at a 3′SS have both been shown to block the second step of splicing^15^,^34^. It remains to be seen whether either of these sequence variations occurs naturally in the context of an RNA that undergoes spliceosomal cleavage as part of its normal biogenesis. The observation that introns are present beyond the 3′ ends of telomerase RNAs in other fungi as well^26^,^35^ suggests a more widely conserved function for the spliceosome in telomerase RNA processing at least in fungi. It will also be important to determine whether telomerase processing is highly unusual or the first example of a group of RNAs for which the spliceosome generates functional products via a single cleavage event. The observation that an intron from the protein-encoding *alp41* gene is efficiently cleaved further supports that spliceosomal cleavage may function as a regulatory mechanism by competing with the completion of splicing, thereby reducing the amount of mature mRNA available for translation.

## Methods

### Yeast strains and constructs

Genotypes of the strains used in this study are listed in Supplementary Table 1. Constructs containing *S. cryophilus*, *S. octosporus* and *A. niger* sequences were generated by replacing the corresponding regions of *Sp*TER1 in pJW10 (ref. 22) with synthetic DNA fragments. Derivatives of these constructs were generated by site-directed mutagenesis and subcloning. Plasmids were introduced into *S. pombe* strains PP138, PP399 and PP407 by electroporation, and transformants were selected on Edinburgh Minimal Media lacking uracil.

### RNA analysis

RNA was isolated from cells grown to a density of 5 × 10^6^ cells ml^–1^ at 32 °C. Cells were collected by centrifugation, washed twice in ddH_2_O, resuspended in ddH2O and quick-frozen by dripping the cell suspension into liquid nitrogen. Cells were lysed in a 6,850 Freezer mill (SPEX SamplePrep) using 8 cycles (2 min) at a rate of 10 per second with 2 min cooling time between cycles. The lysed cell powder was transferred directly into tubes containing 10 ml phenol/chloroform/isoamyl alcohol (25:24:1) and 10 ml sodium acetate (50 mM), 1% (w/v) sodium dodecyl sulphate preheated to 65 °C. RNA was extracted five times with phenol/chloroform/isoamyl alcohol and once with chloroform/isoamyl alcohol. Total RNA was ethanol precipitated and resuspended in 50 mM sodium acetate (pH 5.2). For use in RT–PCR, the RNA was further purified using the RNeasy mini kit (Qiagen) following the manufacturer’s instructions for DNase digestion and RNA cleanup. With the exception of Figs 1a and 5b, all samples used for northern blotting were subjected to RNaseH cleavage prior to gel electrophoresis to enhance resolution of precursor, spliced and cleaved forms. Briefly, 15 μg of DNase-treated RNA were combined with 600 pmol of each of the two DNA oligonucleotides (BLoli1043; 5′- AGGCAGAAGACTCACGTACACTGAC -3′ and BLoli1275; 5′- CGGAAACGGAATTCAGCATGT -3′) complementary to sites in the first and second exon, respectively. The mix was heated to 65 °C in a heat block, and a second heat block at 75 °C was placed on top of the tubes to reduce condensation. After 5 min, the heat-block sandwich was transferred onto a Styrofoam box to allow slow cooling to room temperature. After 45 min, RNaseH buffer New England Biolabs (NEB, final concentration 1 × ) and RNaseH enzyme (5 units) were added and samples were incubated at 37 °C for 30 min. RNaseH-treated samples were ethanol precipitated for at least 1 h at −20 °C. RNA pellets were recovered by centrifugation, dissolved in 1 × formamide loading buffer and run on a polyacrylamide gel for northern analysis as described below.

### Northern blot

For northern analysis, RNA was separated on 4% polyacrylamide gels in Tris-borate–EDTA containing 7 M urea and transferred to a Biodyne nylon membrane (Pall Corporation) in Tris-borate–EDTA buffer. The probe for TER1 was generated by nick-translation of a PCR product in the presence of [α-32P]dCTP (TER1 nucleotides 536–998). *S. octosporus* telomerase RNA was detected by nick-translation of a PCR product obtained with primers BLoli1889 (5′- GGTGAACGCGGTTCCATCTTTCTC -3′) and BLoli1888 (5′- CCCAATATGCAAATTCTCAAATC -3′). Similarly, *S. cryophilus* telomerase RNA was detected using a PCR fragment synthesized with primers BLoli1744 (5′- GTTGGCTGACTATGCTCTGGTCTG -3′) and BLoli1743 (5′- CCCAAATACAAAAGTGTTTCCAGAAGC -3′). Oligonucleotide BLoli1136 (5′- CGCTATTGTATGGGGCCTTTAGATTCTTA -3′) was labelled with polynucleotide kinase in the presence of [γ-32P]ATP to probe for the small nucleolar RNA snR101 (AJ632019) as a loading control. Membranes were hybridized in Church–Gilbert buffer at 55 °C (TER1 probes) and 42 °C (snR101 probe).

### RT–PCR

RT–PCR for TER1 was performed using the following primer pairs: *S. pombe* (BLoli1275, 5′- CGGAAACGGAATTCAGCATGT -3′ and Bloli 1020, 5′- CAAACAATAATGAACGTCCTG -3′); *S. cryophilus* (BLoli1666, 5′- GATCAAAGTTTCGTACTTGTTC -3′ and BLoli 1657, 5′- GTTCGTATCATAATCGTGTG -3′); and *S. octosporus* (BLoli1665, 5′- AGAAGGGGATGCTCTTGTTG -3′ and BLoli1671, 5′- GACATTATGCAGAACTTCCTG -3′). DNase-treated RNA samples (2.5 μg) were combined with oligonucleotides (10 pmol) and dNTP mix (10 nmol) in 13 μl, and samples were heated to 65 °C for 5 min. After cooling, the volume was increased to 20 μl by the addition of RNasin (40 U, Promega), dithiothreitol (DTT) (5 mM final), 5 × first-strand buffer and Superscript III reverse transcriptase (200 U, Invitrogen) as indicated. Samples were incubated at 55 °C for 60 min, RNaseH (5 U, NEB) was added followed by incubation at 37 °C for 20 min. Aliquots (2 μl) of the RT reactions were used for PCR amplification with Taq polymerase (NEB) and oligonucleotide primers under the following conditions: 5 min at 94 °C followed by 30 cycles of 30 s at 94 °C, 30 s at 57 °C and 60 s at 72 °C, followed by 10 min at 72 °C.

For 3′ end sequence analysis, DNase-treated RNA (2.5 μg) was incubated with poly(A) polymerase (600 U, US Biologicals), RNase inhibitor (RNasin, 40 U) and ATP (0.5 mM) in 20 μl reactions at 30 °C for 30 min. The reaction volume was increased to 35.5 μl by the addition of the DNA oligonucleotide PBoli560 (GCGGAATTCT_18_, 125 pmol) and dNTP mix (25 nmol), and the reactions were incubated at 65 °C for 3 min followed by slow cooling to room temperature. The reaction volume was increased to 50 μl with first-strand buffer (Invitrogen), DTT (5 mM), RNasin (40 U) and Superscript III reverse transcriptase (200 U, Invitrogen), and reactions were incubated at 50 °C for 60 min, followed by addition of RNaseH (5 U, NEB) and incubation at 37 °C for 20 min. Aliquots (2.5 μl) were then used in PCR with Taq polymerase (5 U, NEB) and oligonucleotide primers BLoli1006 (5′- CATTTAAGTGCTTGTCAGATCACAACG -3′) and PBoli560 (200 nM each) under the following conditions: 3 min at 94 °C followed by 32 cycles of 30 s at 94 °C, 45 s at 55 °C and 120 s at 72 °C, followed by 7 min at 72 °C. PCR products were separated by electrophoresis on 0.8% agarose gels, and bands of the correct size were excised, purified and cloned into the TOPO TA cloning system (Invitrogen) for sequence analysis.

*S. cryophilus* and *S. octosporus* 5′ and 3′ ends were determined by generating circular RT–PCR products as follows. DNase-treated RNA samples (10 μg) were incubated at 37°C for 1 h with 2.5 units of tobacco acid pyrophosphatase and 40 Units of RNase inhibitor (RNasin) in the presence of 1 × tobacco acid pyrophosphatase buffer. The reaction volume was increased to 120 μl by the addition of ddH_2_0, and RNA was recovered by phenol/chloroform extraction and ethanol precipitation for at least 1 h at −20 °C. Following centrifugation, pellets were dissolved in 12 μl of ddH_2_0 and concentrations determined by nanodrop measurement. RNA samples (4 μg) were incubated with 20 U of T4 RNA ligase (NEB) in 1 × reaction buffer at 16 °C for 16 h, and RNA was recovered by phenol/chloroform extraction and RNA precipitation. RNA pellets were resuspended in 12 μl ddH_2_0. Telomerase RNA was reverse transcribed using JLoli2 (5′- GAAGAGTACGGTTACACGAAG -3′, *S. pombe*), BLoli1653 (5′- CATGGAGAACATCTACTGAGG -3′, *S. cryophilus*) and BLoli1662 (5′- GAGAAGACATTATACTGCAGAC -3′, *S. octosporus*), followed by amplification of fragments containing the site of ligation using BLoli4483 (5′- TACGATTTAGGTGACACTATAGCTAGTAAAATAGTTGACTAC -3′) and JLoli2 for *S. pombe* TER1, BLoli1654 (5′- CTTGTAACCAAGCGTAGTT -3′) and BLoli1653 in case of *S. cryophilus* and BLoli 1665 (5′- AGAAGGGGATGCTCTTGTTG -3′) and BLoli1662 for *S. octosporus*. PCR products were purified and cloned into the TOPO TA vector (Life Technologies) for sequence analysis.

### Telomerase activity assay

*S. pombe* cultures (1.5 l) were collected at 5 × 10^6^ cells ml^–1^, washed in TMG(300) (10 mM Tris-HCl buffer, pH 8.0, 1 mM magnesium acetate, 10% (v/v) glycerol, 300 mM sodium acetate), resuspended in one packed cell volume TMG(300) plus supplements (5 μg ml^–1^ chymostatin, 5 μg ml^–1^ leupeptin, 1 μg ml^–1^ pepstatin, 1 mM benzamidine, 1 mM DTT, 1 mM EDTA and 0.5 mM phenylmethylsulphonyl fluoride) and quick-frozen by dripping into liquid nitrogen. Cells were lysed in a 6,850 Freezer mill (SPEX SamplePrep) using six cycles (2 min) at a rate of 10 per second with 2 min cooling time between cycles. After thawing on ice, two packed cell volumes of TMG(300) plus supplements were added. All subsequent steps were perfomed at 4 °C. Extracts were cleared by two rounds of centrifugation at 14,000*g* for 10 min and frozen in liquid nitrogen for storage at –80 °C. Telomerase was enriched on agarose beads coated with anti-c-Myc (9E10, Santa Cruz), and telomerase activity assays contained 10 μl of beads in 10 μl of 50 mM Tris-acetate, pH 8.0, 100 mM potassium acetate, 1 mM magnesium acetate, 5% (v/v) glycerol, 1 mM spermidine, 1 mM DTT, 0.2 mM dATP, dCTP and dTTP, 2 μM [α-32P]dGTP (500 Ci mmol^–1^) and 5 μM primer (PBoli14, 5′- TGTGGTGTGTGGGTGTG -3′). Reactions were incubated at 30 °C for 90 min and reaction products analysed on a 10% polyacryl amide gel.

## Author contributions

R.K. and P.B. designed the overall study. R.K. performed the experiments except for the initial characterization of TER1 RNA from *S. cryophilus* and *S. octosporus*, which was carried out by R.M.H., Wen Tang, Jessica Box and Jeremy Bunch. Data shown in Fig. 4c (P.B.), and Fig. 5a and Supplementary Figs 4 and 5 (R.M.H.). Computational analysis of introns was performed by R.O.D. R.K. and P.B. analysed the data and wrote the manuscript.

## Additional information

**How to cite this article:** Kannan, R. *et al*. Diverse mechanisms for spliceosome-mediated 3′ end processing of telomerase RNA. *Nat. Commun.* 6:6104 doi: 10.1038/ncomms7104 (2015).

## Supplementary Material

Supplementary Information Supplementary Figures 1-5, Supplementary Table 1 and Supplementary References.
