# Supplementary material for: Diverse mechanisms for spliceosome-mediated 3′ end processing of telomerase RNA
Source: Nat Commun. 2015 Jan 19;6:6104. doi: 10.1038/ncomms7104 (PMC4299874; doi:10.1038/ncomms7104)
Supplement: Supplementary Information — Supplementary Figures 1-5, Supplementary Table 1 and Supplementary References. [file ncomms7104-s1.pdf]

A

+1

AAACAAGAACCAGAAUCCCAGUUUCAAGAAGAAUUAUGAGGAGUUUGGUUUUAUGAUUCUUUAGGGCUUA

10 76 12 11

CAUGGAAAGUCGAAUAACGCAAGGAAGAAAAGUUUGAAUGGUGUUUAUGAUUUGAUGUUUGCAGUAUACU

GUCUUCUUAGUAUACAAAGGGAUUGAGGAUGAAAAAGGAAAAUCCUUUUCUCAAACCUCAUUUGUUGGAC

GGGACGUCCAGCAACCACUUUGUGUAACCCAAGUAUUCUUUUUAUAUUCAGUGCUGCGAAGGAAAAGGUG

AUCAAAAGGUAAAGAAUACUAGAUCUUUUUUUGUGUCUUUACAGUUUCCUCAGUAGAUGUUCUCCAUGGA

AUCACUGUACCCUUGGAUGAAGUCUGUUAUAAAUUUGAUCUCUCAAACCAUUUUCAUGACUUGCAGUAGU

AUUGUAUGGUCCUUUGAGGGAGGCAUUGAGAGAGCCUGGAUACCAAUUGUACUGCCGGGUUGGCAGAUGU

GGAUCUUCUGCUUUAUCAGUGUUUUUUUCUUAGCUUUGGUGUUUUUAUGCCUGGCUAGUUCUCGAGAGAA

AUACGAUGGCUAUUGCAGGGCUUGAAAAUGACUCGCACAGAUCGCAGUGUAUUUUUUGCAGGAGGCAUUC

UUUGAUUUGAGUGAGAAUGAAAUGACAGAAAAAAGGUGCCUUUUUGUUGCUUUACUUUUCAGCCUACUCU

CAAGGUUCAUAAAUAGGAUCUUGUAGUAGCAACAAGUGUUUCUUUACAUAAUUUGUAAAGAAACAUACG

UUGGCUGACUAUGCUCUGGUCUGUAGAAUUUUUAUUCUAUGGAAGCUUAGAGACGUAUUGAUUCCAGGUU

UUCUACAAACCCAUUUUGGCUUUUUUGUUGGUGUUUUGACCGUGCCCCUUGUAUUCUUCAGGUGAAGGUG

CAAGCAGGAAAGAAUUAAAAACCAAUUGGUUGACACAAUGUACUUUCCAACAAUUGUAGAUACAGUUCCU

UCUUUUCAAUGGCUCUUCACAGUUGUUGUCGAACGAAACUACUUGCUAUUUAGCUUUUCUUGUAACCAAG

CGUAGUUGGGAAGAAAGGAGGGAAAUUUGAUGUACCGAGAGUCACCAAUUCUAGCUCUUUUUAUAACUUU

AGGAGAUCCAAAUGAUCAAAGUUUCGUACUUGUUCCUGACUUUAUGAAAAAAGUUAGCUGGUAGUCCUUG

AUUCAUUCAGGUUUAUUUCUGACACCCGACUAUUGUCCCUACAGAGUGCAUAUUGCCAAAUUUUCUUCAG

2 9 24 23 12 3 1 11 1 1

CUUCUGGAAACACUUUUGUAUUUGGGAAAUUUUUUUUUCCUUGCUUCCGgucagugcuacuauguuuucu

* * *

uucgcgacuaacagaaucuuuguagUUUUGUUAAUCACACGAUUAUGAUACGAACGCUUUUAUUCAAAAA

1

CAAGAUUAAAAAGGUGUAUUAAUCGAUGGCACGUCUUUUAAAAAUUAAUUAAUAAAGCUUAUAAUAAAAG

1 4 5

UCUAUUUAUGAAUCAUGAA 1484

Kannan, et al.; Suppl. Figure 1A

B

+1

AAACAUGAACUUGAAUCCCAGUUGGAAGAAGAAUCGCAUGGAGGUUGGUUUUAUGAUUCUUUAGGGCUUA

10 64 13 1

CGUGAAACUUCUAAAGAAAAAAGAGGAAACUUGUGAACCGCGUUUACUAUUUCAUGUCUGCAGUAUAAUG

UCUUCUCAGUAUACAAAAAGGUAAAAGUAAUGGAAAAAGGGAAUCCUAUUUCCAAACUUCAUUUGCUAGA

CGGGACGGCUAGCAGACUUUUGUGUAACCCAAGUAUUCUUUUCAUAUUCAGCGCUGUGAAGGAAGAGUAU

CAAGAGAUAAAAUAUGCAGUAUUUUUUUUCUGUCUUUCCACUUUGCUCAGCAGAUGCUCUCCAUGGAAUC

ACUGUACCUUGGUGGGCUGUUAUUAAGGCCAAUCUUUCUAACCACUUAUUCAAUGAGUACGAUAGUAUGU

UGUACUUUCCUGUGAGAGACAAUGAGGAAGCUUGGAUACCAAUUGUACUGCCGAAUUUGGUGAAUGUGGA

UCUUCUGCUUCAUCAUUGUUUGUCUGGCCUGGUAUAUCUCAAGGAAGGUAUAACGACUGUUUGUAGGAUU

UGGAAGUGACUUAUGCAGAUAGCAGUGUAUUUUUUGUAGGAGAGUUUCUUUGACCUGAGUGAGUGUGAUU

UUCAGAGAAAGAAUCUCUUUUGUCGCUUUCCUUUCCCAGCCUUCUCAAGUUUCGCUAGUAUGUAGAAAUU

UGCAGAAGCAAUAGACAUCCUUGGUCUAUCCUGGUUUGAGGAUAGCAAUUGGCUGAUUACGCUCUGGUCU

AUGGAAUUUUAUAUUCUAUAGAAGCUUAGAGAUUCUACUGAUAUCAACUCUGAACAGUAUUUUGGCUUUU

UUGUCGGUAUUUUGACCGUGCCGCUUGUAAUCUUCAUGCGAAGAAGAUUCAUGCAGGAAAGAAUCAAAAA

UCGACUAGUUGACACAAUGUACUUUCCAACAAUGGUGAACGCGGUUCCAUCUUUCUCAAAAGUGAAAACA

CUCGUGUAGAGUGGGACUUCUUUAUUUCUUAGUUUUUCAUGUAAUCAAGCGUAGUUAGAAAAAAAAGAGA

AGGGGAUGCUCUUGUUGUUUUAGAAAUUGCCAAUCUUGUUUUUUUAUAAAGGAAUGAGAUCCAACGUGAU

CAAAGUUUCGUACUUGUUCAUUCCGUUAUAAAAAGAUUAAGUAGGCAAUGUUCACUUCACUUGGGAUCCU

1

CUUUUCAUACUAACUAUUGUCCCUAUAGAGUGCAUAUUACCAACCCUACUUUAGAUUUGAGAAUUUGCAU

1 1 2 17 23 22 15 3 1 5

AUUGGGAAUUUUUUUUCUCUCUCUUUUUACUAUACUUAUCGgucagugcuauuauguuuucaacguggcu

* * * * *

aauguucuuguuuuagUUCUUGGAAGCCCAAAGUACAUUAUAAUACAUCACCUUUUGUUCGAGUCAUGCU

UGAAAAUUAAAGGAAACUCUAAAACAGGAAGUUCUGCAUAAUGUCUCCUAAAUUUGAGAAAUAAAGAGCA

1

GAUGCGAUUUUACUUAACUCAAGGCAUAAAGUUUAUGGUCAUAA 1510

Kannan, et al.; Suppl. Figure 1B

Supplementary Figure 1: Annotated TER1 sequences for **(A)** *S. cryophilus* and **(B)** *S. octosporus*. Primers BLoli1110 (TGCTCT[S]TATGGTTGGATGGAC) and BLoli1113 (TCTCC[W]ACGAC[S]AC[M]AGTTTCTT), corresponding to conserved regions in flanking genes, were used to amplify the TER1 gene from genomic DNA samples of *S. cryophilus* and *S. octosporus*. 5' and 3' ends of the RNA were determined by circular RT-PCR. Starting positions are indicated by horizontal arrows, 3' ends by vertical arrows, and numbers represent the cloned isolates that start or end at that position, respectively. All 3' ends downstream of the introns were poly-adenylated. The template region is underlined, the putative Sm/LSm binding site is boxed and the introns are shown in lower case. Six (*S. cryophilus*) and 12 (*S. octosporus*) circular RT-PCR clones were excluded from the figure due to ambiguity regarding the precise point of ligation. One to five non-templated adenosines were found at the 3' end of 13 (*S. cryophilus*) and 38 (*S. octosporus*) clones. The positions are indicated with asterisks.

Telomere C1

GGGTTACAAGGTTACGTGGTTACACGGTTACAGGGTTACAGGTTACAGGGGGGTTACAGGTTACGGTTACAGGG
GTTACTTGGGTTACATTTTACTTGGGTTACTTGGGTTACTTGGGTTACTTGGGTTACTTGGGTTACTTGGGTTACTT
GGGTTACT

Telomere F1

GGGTTACAAGGTTACGTGGTTACACGGTTACAGGGTTACAGGTTACAGGGGGGTTACAGGTTACGGTTACTTGGG
TTACTTGGGTTACTTGGGTTACTTGGGTTACTTGGGTTACTTGGGTTACTTGGGTTACTTGGGTTACTTGGGTTACT
TGGGTTACTTGGGTTACTTGGGTTACTTGGGTTA

Telomere A7

GGGTTACAAGGTTACGTGGTTACACGGTTACAGGTTACAGGTTACAGGGGGGTTACAGGTTACGGTTACAGGGG
TTACTTGGGTTACTTGGGTTACTTGGGTTACTTGGGTTACTTGGGTTACTTGGGTTACTTGGGTTACTTGGGTTACA
CTTGGGTTACTTGGGTTACATTTACTTGGGTTACTTGGGTTACTTGGGTTACTTGGGTTACTTGGGTTAC

Telomere H8

GGGTTACAAGGTTACGTGGTTACACGGTTACAGGTTACAGGTTACAGGGGGGTTACAGGTTACGGTTACAGGGG
TTACGGTTACAGGGTTACTTGGGTTACTTGGGTTACTTGGGTTACTTGGGTTACTTGGGTTACTTGGGTTACTTGG
GTTACTTGGGTTACATTTGCTTGGGTTACTTGGGTTACATTTTGCTTGGGTTACTTGGGTTACTTGGGTTACTTGGG
GTTACTTGGGTTACTTGGGTTACTTGGGTT

Telomere H11

GGGTTACAAGGTTACGTGGTTACACGGTTACAGGGGTTACACGGTTACAGTTACAGGTTACAGTTACAGTTACAG
GTTACGGTTACGGTTACAGGGTTACGGTTACAGGGTTACAGGTTACAGGTTACAGGGGGGTTACAGGGTTACTTG
GGTTACTTGGGTTACTTGGGTTACTTGGGTTACTTGGGTTACTTGGGTTACTTGGGTTACTTGGGTTTACTTGGGTT
ACTTGGGTTACTTGGGTTACTTGGGTTACTTGGGTTACTTGGGTTACATTGGGTTACTTGGGTTACTTGGGTTACTT

Supplementary Figure 2: Telomere sequences cloned from *S. pombe* cells expressing TER1 with the template region of *S. cryophilus* TER1. The proximal part of each telomere comprised of original *S. pombe* repeats is shown in green; *S. cryophilus* repeats are shown alternately in red and blue. Regions where the sequence deviates from perfect GGGTTACTT repeats are underlined. Five telomeres representative of 63 cloned telomeres are shown.

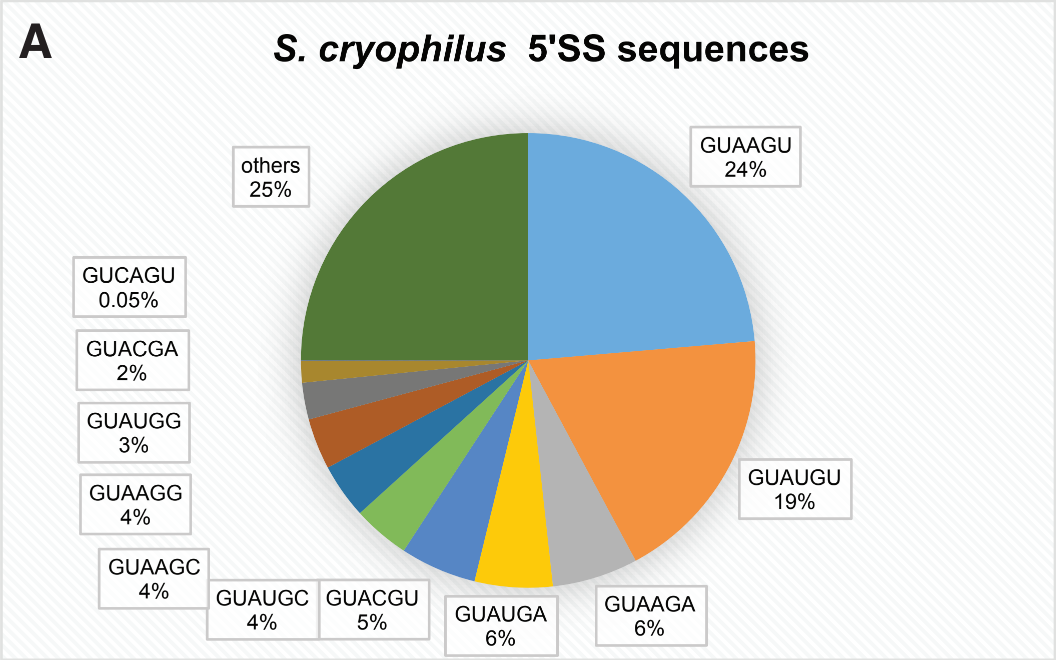

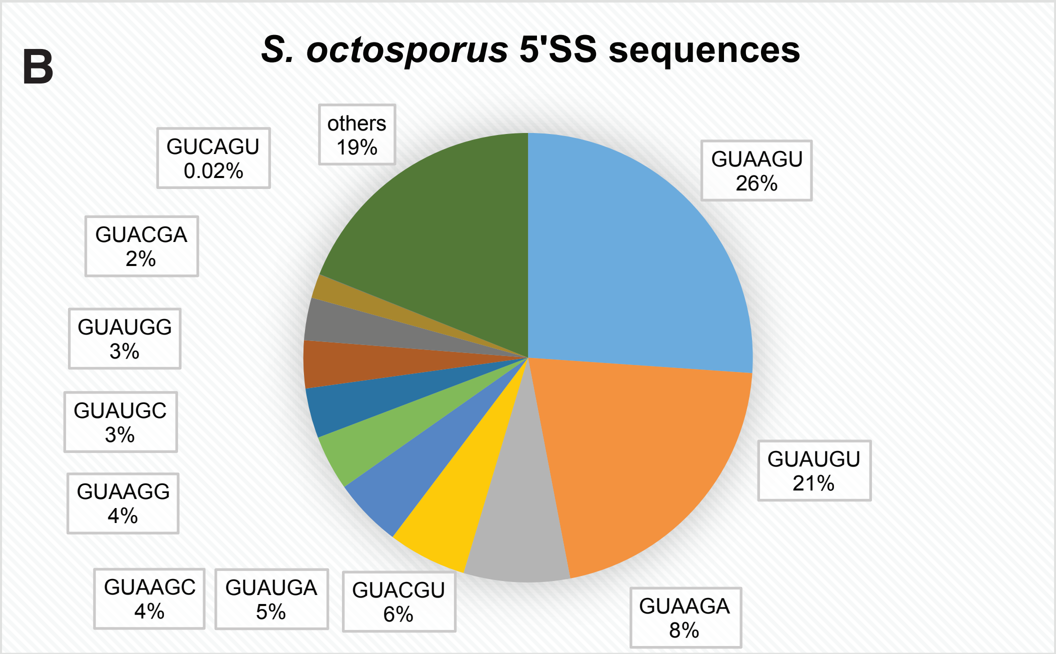

Supplementary Figure 3: Distribution of 5' splice site sequences in (A) *S. cryophilus* and (B) *S. octosporus*. The first six nucleotides of all annotated introns were used for the analysis. Sequences that make up less than 2% of the total are summarized in the “other” category except for the GUCAGU sequence.

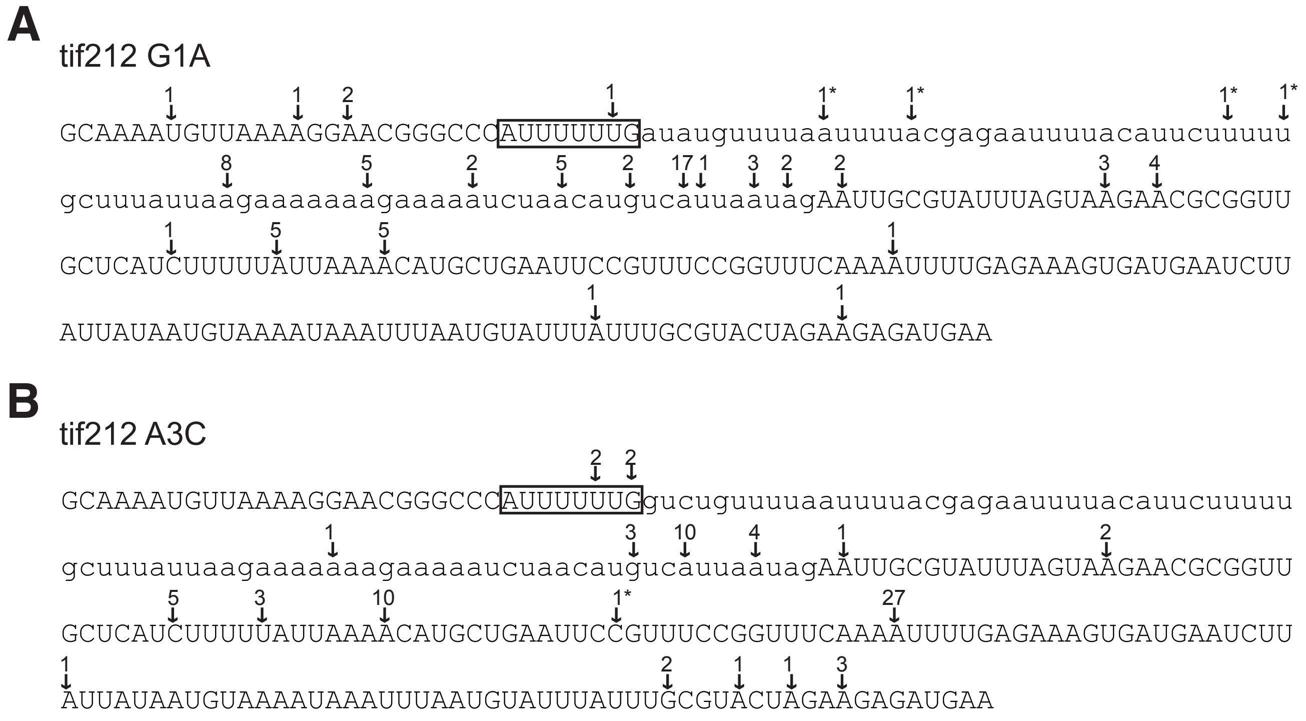

Supplementary Figure 4: Annotated sequences of the 3' end of TER1 containing **(A)** tif212 G1A and **(B)** tif212 A3C introns. Ends were mapped by cloning and sequencing of circular RT-PCR products. Vertical arrows indicate 3' ends and numbers represent the clones ending at that position. All termini downstream of the Sm/LSm site were poly-adenylated, except where indicated by an asterisk. The Sm/LSm binding site is boxed and the introns are shown in lower case.

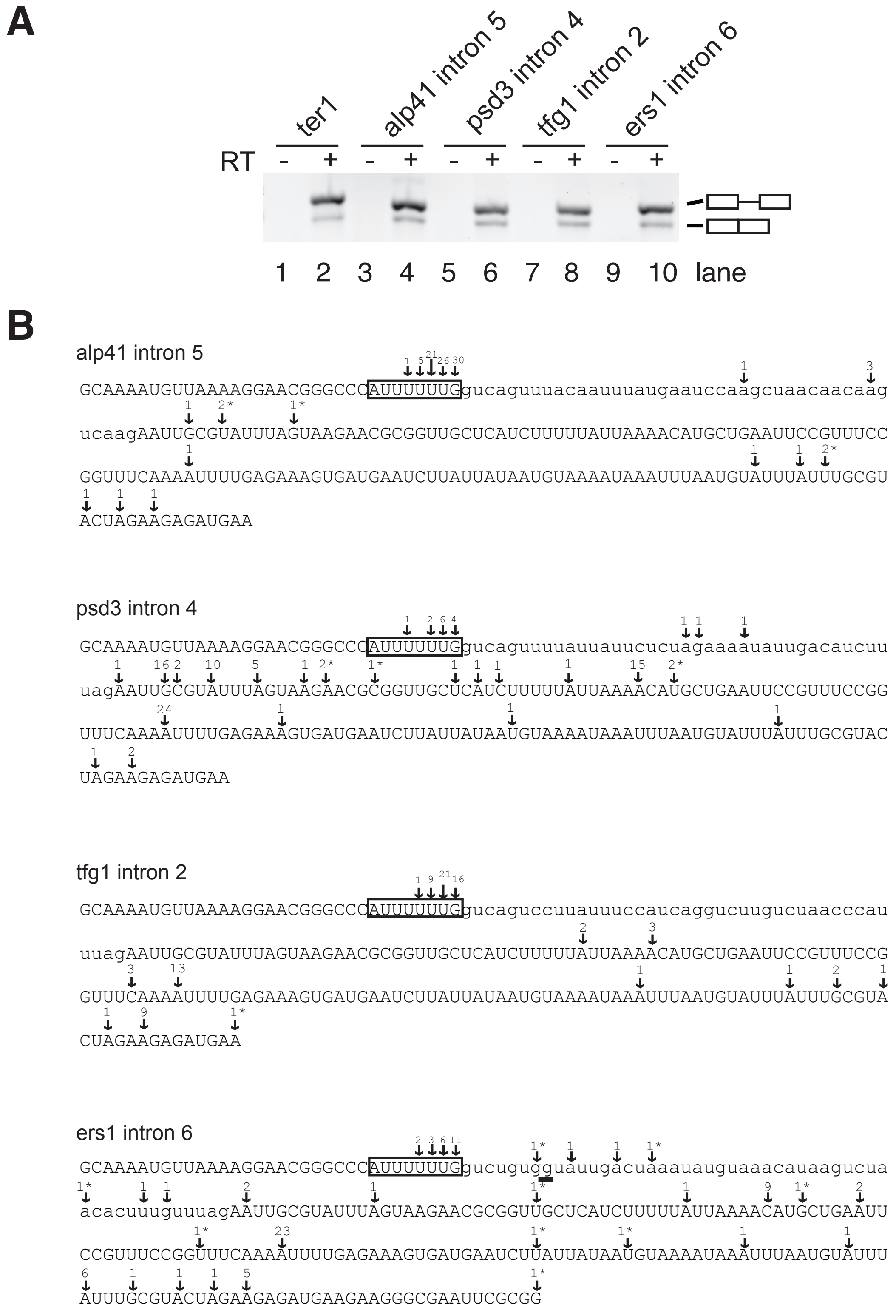

Kannan, et al; Suppl. Figure 5

Supplementary Figure 5: Processing of TER1 transcripts containing introns from protein encoding genes with non-canonical 5'SS. (A) RT-PCR across intron. **(B)** Annotated sequences of the 3' end of TER1 containing alp41 intron 5, psd3 intron 4, tfg1 intron 2 and ers1 intron 6, respectively. Ends were determined by circular RT-PCR and are indicated by vertical arrows, with numbers representing clones ending at that position. All sites downstream of the Sm/Lsm site were poly-adenylated, except where indicated by an asterisk. One, eight, six and eight spliced clones were observed for the alp41, psd3, tfg1 and ers1 introns, respectively. For the ers1 intron one clone used an alternative 5'SS, position +1 of which is underlined. The Sm/Lsm binding site is boxed and the introns are shown in lower case.

Supplementary Table I

Genotypes and sources of strains used in this study

Strain Number	Genotype	Source
PP138	*h⁻ ade6-M216 leu1-32 ura4-D18 his3-D1*	Lab stock
PP265	h⁻	Lab stock
PP399	leu1-32 ura4-D18 his3-D1 trt1-Cmyc9 ter::Kan^R	Lab stock
PP407	*h⁺/h⁻ leu1-32/leu1-32 ura4-D18/ura4-D18 his3-D1/ his3-D1 ade6-M210/ade6-M216 ter1⁺/ ter1::kanMX6*	ref [1]

Species	Origin	Source
S. cryophilus	as described in [2]	Lab stock
S. octosporus	CBS-KNAW Fungal Biodiversity Centre	Lab stock based on CBS371

Supplementary References

1 Box, J. A., Bunch, J. T., Tang, W. & Baumann, P. Spliceosomal cleavage generates the 3' end of telomerase RNA. *Nature* **456**, 910-914 (2008).

2 Helston, R. M., Box, J. A., Tang, W. & Baumann, P. Schizosaccharomyces cryophilus sp. nov., a new species of fission yeast. *FEMS Yeast Res* **10**, 779-786 (2010).
